# Brain response in asthma: the role of “lung-brain” axis mediated by neuroimmune crosstalk

**DOI:** 10.3389/fimmu.2023.1240248

**Published:** 2023-08-24

**Authors:** Yao Wang, Ya-Kui Mou, Han-Rui Wang, Xiao-Yu Song, Shi-Zhuang Wei, Chao Ren, Xi-Cheng Song

**Affiliations:** ^1^ Department of Otorhinolaryngology, Head and Neck Surgery, Yantai Yuhuangding Hospital, Qingdao University, Yantai, China; ^2^ Shandong Provincial Clinical Research Center for Otorhinolaryngologic Diseases, Yantai Yuhuangding Hospital, Yantai, China; ^3^ Yantai Key Laboratory of Otorhinolaryngologic Diseases, Yantai Yuhuangding Hospital, Yantai, China; ^4^ Shandong Provincial Innovation and Practice Base for Postdoctors, Yantai Yuhuangding Hospital, Yantai, China; ^5^ Department of Neurology, Yantai Yuhuangding Hospital, Qingdao University, Yantai, China

**Keywords:** asthma, brain response, lung-brain axis, neuro-immune, inflammatory response

## Abstract

In addition to typical respiratory symptoms, patients with asthma are frequently accompanied by cognitive decline, mood disorders (anxiety and depression), sleep disorders, olfactory disorders, and other brain response manifestations, all of which worsen asthma symptoms, form a vicious cycle, and exacerbate the burden on families and society. Therefore, studying the mechanism of neurological symptoms in patients with asthma is necessary to identify the appropriate preventative and therapeutic measures. In order to provide a comprehensive reference for related research, we compiled the pertinent literature, systematically summarized the latest research progress of asthma and its brain response, and attempted to reveal the possible “lung–brain” crosstalk mechanism and treatment methods at the onset of asthma, which will promote more related research to provide asthmatic patients with neurological symptoms new hope.

## 1 Introduction

Asthma, also known as bronchial asthma, is a chronic inflammatory disease of the airways with a complex etiology, and its pathological process involves multiple aspects, such as the genetic regulation of genes and environmental interference (1, 2). According to a 2022 study, over 334 million individuals are suffering from asthma worldwide, with an incidence of approximately 3.33%, which had risen rapidly because of the synergistic influence of air pollution, climate change, and other factors over the last few decades (3, 4). Asthma primarily affects children and adolescents, and has become a severe global public health issue, imposing a significant economic burden on families and society (5).

Asthma is characterized by non-specific respiratory symptoms such as wheezing, shortness of breath, chest tightness, and episodic cough as the main clinical manifestations, which frequently occur at night and early in the morning, and its main pathogenesis is chronic immune-inflammatory response, airway hyperreactivity, reversible airflow limitation, and airway remodeling (6, 7). Patients with asthma are often accompanied by neurological symptoms such as cognitive dysfunction, depression, anxiety, dysosmia, and sleep disorders, implying a brain response to asthma, which affects their quality of life, increases their economic burden, reduces the treatment sensitivity of asthma, increases the risk of asthma exacerbations, and forms a vicious cycle (8–10). Based on our previous summary of brain response in allergic rhinitis (AR) (11), a “lung–brain” crosstalk in asthma can be observed on the basis of neuro-immune mechanisms, that is, inflammatory factors generated during chronic inflammation in asthma can be transmitted upwards to the central nervous system, thereby stimulating associated brain regions to elicit one or more brain responses, transmitting response commands to peripheral nerves, activating such commands to release mediators such as neuropeptides and neurogenic trophic factors, worsening asthma symptoms through actions such as tracheal smooth muscle contraction, promoting the re-entry of immune inflammatory factors produced in the periphery to the brain, and exacerbating neurological symptoms caused by brain response (12, 13). We have summarized the research on the correlation between asthma and changes in brain activity related to its neurological phenotypes in the past 5 years (**Table 1**) (14–22).

**Table 1:** Summary of research on the correlation between asthma and changes in brain activity related to its relatedneuropsycological symptoms in the past 5 years.

Sample	Age (asthma/control)	Numbers (Asthma)	Methods of brain function detection	Indicators of brain function detection	Associated neurological phenotypes	Evaluation methods of neurological phenotypes	Conclusions	References
Children	8.98 ± 1.52/8.31 ± 1.39	62 (31)	fMRI	DC and VMHC	Cognitive dysfunction	CPT	Impaired superior frontal gyrus and parietal lobe function are associated with attentional deficit in asthmatic children.	Zhu L, et al. (14)
Adults	39.58 ± 2.19/38.53 ± 1.70	38 (19)	fMRI	DMN and SN	Depression and anxiety disorders	DASS	Enhancements in power and coherent activity of DMN and SN regions, including dmPFC, vmPFC, PCC, rACC, precuneus, parietal cortex, insula, and dACC in asthmatic patients, that were remarkably correlated with depression, anxiety.	Gholami-Mahtaj L, et al. (15)
	18-73	246 (111)	dMRI	DWI and MD	Cognitive dysfunction	Reaction time of the Stroop Task	Deterioration of myelin axons of fiber bundles of the corticospinal tract, external capsule, inferior longitudinal fasciculi, superior longitudinal fasciculi, inferior fronto-occipital fasciculi and the destruction of white matter integrity is related to the cognitive dysfunction.	Rosenkranz MA, et al. (16)
	52.2 ± 9.70/50.43 ± 9.42	80 (40)	fMRI	SN, DMN and ECN	Depression disorders	HAMD	The functional connection between dACC and the left middle frontal gyrus increases, as well as the functional connection between SN and DMN, ECN, which leads to depression of patients with asthma.	Zhang Y, et al. (17)
	18-45	40 (40)	fMRI	Subcortical gray matter volumes	Depression and anxiety disorders	HADS	The strong anxiety of patients with asthma is related to the lower volume of the pallidum, while general anxiety and depression are not significantly correlated with subcortical gray matter volume.	Ritz T, et al. (18)
	38.67 ± 10.46/38.64 ± 10.35	98 (54)	fMRI	The global and regional network measures for brain DTI networks	Depression and anxiety disorders	HRSD and HRSA	The abnormal nodal centralities that involved the fronto-limbic network and the right middle temporal gyrus (temporal pole) is associated with depression and anxiety in patients with asthma.	Gao X, et al. (19)
	18-55	20 (20)	fMRI	BOLD	Depression and anxiety disorders	HADS	Deteriorating asthma symptoms are associated with negative emotional stimuli and stronger activation of the anterior and middle cingulate gyri, including the dACC.	Ritz T, et al. (20)
	25.3 ± 8.9/25.10 ± 8.88	40 (20)	sMRI, ^1^H-MRS	Hippocampal volume and metabolites	Cognitive dysfunction	MoCA	Cognitive impairment in patients with asthma is related to the reduction of NAA and glutamate Glu in the hippocampus, but not to the volume of the hippocampus.	Kroll JL, et al. (21)
	31-62	89 (32)	pASL, fMRI	CBF	Depression disorders	HRSD	Depression disorders in patients with asthma is associated with increased rCBF in the right cerebellum posterior lobe.	Zhang Y, et al. (22)

fMRI, functional magnetic resonance imaging; Dmri, diffusion-weighted magnetic resonance imaging; sMRI, structural magnetic resonance imaging; ^1^H-MRS, proton magnetic resonance spectroscopy; pASL, pulsed arterial spin labeling; DC, degree centricity; VMHC, voxel-mirrored homotopic connectivity; DMN, the default mode network; SN, the salience network; DWI, diffusion-weighted imaging; MD, mean diffusivity; ECN, executive control network; DTI, diffusion tensor imaging; BOLD, blood oxygenation level dependent; CBF, cerebral blood flow; CPT, continue performance test; DASS, Depression Anxiety Stress Scales; HAMD, Hamilton depression scale; HADS, Hospital Anxiety and Depression Scale; HRSD and HRSA, Hamilton Rating Scale for Depression and Anxiety; MoCA, the Montreal Cognitive Assessment; vmPFC, ventromedial prefrontal cortex; dmPFC, dorsomedial prefrontal cortex; PCC, posterior cingulate cortex; ACC, anterior cingulate cortex; NAA, N-acetylaspartate; Glu, glutamate; rCBF, regional CBF.

Despite the abundance of research on asthma and neuroinflammation, the specific brain response mechanism implicated in asthma has not been systematically elucidated. Thus, we aim to sort out the current status and progression of neurological symptoms induced by asthma by summarizing relevant literature to provide a reference for mechanism exploration, prevention, and treatment intervention of asthma-related brain responses. Simultaneously, our summary also complements the “lung–brain” axis theory to a certain extent.

## 2 Asthma and neuro-immune-mediated “lung–brain” crosstalk

Patients with asthma exhibit altered functional activity in relevant brain regions. Using resting-state functional magnetic resonance imaging (fMRI), Huang et al. scanned the brain regions of patients with asthma and found differences in regional homogeneity values in the cerebellar, frontal, temporal, and occipital lobes (23). Rosenkranz et al. used fMRI to simultaneously measure pulmonary function and inflammatory parameters of induced sputum in patients with asthma and found that changes in insula activity best characterized asthma pathogenesis; such changes were significantly associated with decreases in pulmonary function and increases in eosinophil content of induced sputum (24). Leupoldt et al. used fMRI to investigate changes in brain regions during induced asthma in asthmatic patients and healthy subjects and found that cortical activity in the insula decreased, whereas activity in the periaqueductal gray (PAG) increased when asthma was induced and caused dyspnea. The authors hypothesized that the activity of the insula, a key brain region involved in emotion regulation, may be regulated by PAG, which causes negative mood disorders such as anxiety and depression (25). Dynamic functional connectivity is more sensitive than resting brain function evaluation indicators in MRI, allowing it to disclose the internal heterogeneity and dynamic changes of brain dysfunction (26), of which dynamic voxel-mirrored homotopic connection (dVMHC) plays a role in revealing asthma-induced brain response because of its sensitive and specific quantitative characteristics of neural activity intensity, particularly in indicating emotion-related disorders (27). A clinical study of patients with asthma revealed that dVMHC levels in the lingual gyrus and calcarine sulcus were significantly elevated, and dVMHC values in the medial superior frontal gyrus, cingulate gyrus, and supplementary motor area were considerably lowered; these brain regions have a vital function in mood regulation, indicating that the ability of patients with asthma to regulate their emotions is severely impaired (28). Thus, a “lung–brain” crosstalk based on the neuro-immune mechanisms may be observed when asthma occurs.

### 2.1 Effects of airway inflammation on the brain in patients with asthma

Prior research on neuro-immune crosstalk in asthma has focused on interactions between inflammatory factors and the peripheral nervous system. Given the increasing number of patients with asthma who develop a variety of neurological symptoms in clinical practice, pertinent research has been conducted, and results show that numerous inflammatory factors produced during chronic asthmatic inflammation can reach the central nervous system via humoral and neural pathways.

Asthma-induced chronic inflammation can stimulate the synthesis and release of prostaglandin mediators and nitric oxide (NO) from cerebrovascular endothelial cells and pericytes that form the blood–brain barrier (BBB), causing endothelial cell injury, tight junction destruction, and structural integrity disruption of the BBB. It can also increase BBB permeability in response to the binding of inflammatory factors to endothelial cell surface receptors (29). In the humoral pathway, after being released into the blood, cellular inflammatory factors can either penetrate the BBB or infiltrate the brain region directly via the periventricular organs without a BBB, thereby activating and damaging neurons, including microglia and astrocytes. Michelle and Parajuli et al. discovered that after eosinophil activation, eotaxin-1 (CCL11) swiftly crossed the BBB and bound to CCL11 receptors on the surface of microglia (30), which increased the amount of nicotinamide adenine dinucleotide phosphate-oxidase 1 (NOX1) in microglia, promoted the development of oxidative stress in microglia, increased the levels of intracellular reactive oxygen species (ROS), and enhanced glutamate-induced neurotoxicity, thereby resulting in neuronal damage and death (31). The pathogenesis of asthma is significantly influenced by oxidative stress. Florentino et al. demonstrated that oxidative stress-mediated inflammation promoted the activation of relevant microglia and the production of inflammatory neuro-mediators after compromising the integrity of the BBB, leading to a series of brain responses (32, 33). In addition, Antunes et al. found that airway inflammation could increase acetylcholinesterase (AchE) activity and decrease Na+, K+-ATPase activity in the brain of asthmatic mice, both of which led to a reduction in acetylcholine (Ach) content and formed a pro-inflammatory humoral immune microenvironment (34, 35).

With regard to neural pathway transmission, after the inflammatory response during asthma attacks activates primary afferent nerves, signals are further emitted to stimulate afferent nerve fibers of the pulmonary vagus nerve, which produce nerve impulses that reach the nucleus tractus solitarius (NTS) and project to multiple brain regions, thereby inducing diverse neurological phenotypes by affecting the functional activity of these brain regions (36, 37). The research of Chen et al. on asthmatic monkeys indicated that protracted exposure to allergens elevated excitability in relevant brain regions (e.g., NTS) that process airway receptor afferent signals, which may be associated with an increase in the plasticity of postsynaptic neurons caused by variations in the amplitude and frequency of nerve impulses transmitted to the NTS by lung receptors (28). Simultaneously, chronic inflammation in asthma increases airway resistance; enhances respiratory reflexes, chemoreceptors, and pulmonary stretch receptors in airways associated with shortness of breath and dyspnea-transmitted nerve impulses to the insula and anterior cingulate cortex (ACC) via the spinothalamic cortex; and completes the regulation of respiration via functional connections formed by the medial prefrontal cortex (mPFC) and brainstem (12, 38). When examining respiratory-related evoked potentials in children with life-threatening asthma (LTA), Davenport et al. found that certain children with LTA had a loss of airway mechanoreceptor-cerebral cortex perception-related circuits, which made their asthma symptoms more difficult to control compared with those of other children (39). The neural pathways implicated in the “lung–brain” axis associated with asthma are intricate and delicate, and they may involve a single brain region, multiple brain regions, or neural circuits, which coordinate and collaborate with humoral pathways to contribute jointly to the effects of peripheral airway inflammation on the brain.

### 2.2 Brain regulation of airways in asthma

Chronic inflammation resulting from the pathogenesis of asthma is transmitted to the central nervous system via a number of pathways, resulting in the activation of related brain regions and subsequent alterations in brain function and symptoms, during which brain signals are fed back to the periphery in various ways. Asthma occurs when the bronchial smooth muscle contracts and generates various inflammatory factors, triggering an inflammatory response in related brain regions to activate its function, including the amygdala, prefrontal cortex (PFC), and insula. Amygdala stimulation further activates the hypothalamic–pituitary–adrenal (HPA) axis, and glucocorticoids are released into the circulation to bind to glucocorticoid receptors (GR) on the surface of the airway epithelium and immune cells as well as to achieve the central regulation of peripheral inflammation, particularly in the expression of cellular inflammatory factors (such as IL-4, IL-5, and IL-13) involved in the development of asthma (12, 40). The activation of the HPA axis can also lead to the release of non-glucocorticoids (e.g., epinephrine, norepinephrine, and acetylcholine), which, along with glucocorticoids, amplify immune inflammation generated by asthma (41). Bailey et al. identified that the expression level of GR in the lungs of stressed asthmatic mice was drastically downregulated following the activation of the HPA axis, which may be connected to the impaired nuclear translocation of GR in macrophages and other cells, thereby reducing the responsiveness of the body to glucocorticoids, suppressing its anti-inflammatory ability, and deteriorating the airway inflammatory response in patients with asthma (42, 43). Similarly, Pongratz et al. suggested that the hypothalamus triggered the sympathetic nervous system to release norepinephrine, which acts directly on B-cell surface-associated receptors to promote the synthesis and release of immunoglobulin E (IgE), a key factor in asthma (44, 45). Therefore, the distinctive endocrine activation route of the HPA axis is crucial to brain’s modulation of peripheral inflammation.

Furthermore, numerous studies have confirmed that asthma is triggered by psychological stress, negative emotions, and other states (46). After induction, patients experience the activation of brain regions, including the ACC, insula, and limbic system, releasing neurotransmitter molecules represented by substance P(SP), histamine, and neuropeptide Y (NPY) that are transmitted to the airways through specific pathways; interacting with peripheral nerves and immune cells to exacerbate asthma symptoms; and deteriorating asthma symptoms to aggravate the adverse emotions of patients with asthma (24, 47, 48). Miyasaka et al. injected histamine receptor (H1R and H2R) antagonists into the brain ventricles of stressed asthmatic mice and discovered a significant reduction in peripheral airway inflammation, and eosinophil and lymphocyte infiltrations were considerably lowered, which indicated that allergens under stress may increase the severity of the inflammatory response to asthma by stimulating the release of histamine to activate histamine receptors (H1R and H2R) in the brain region (49). Consequently, the regulation of the expression of these neurotransmitter molecules can be used as an effective means to manage asthma symptoms.

## 3 Asthma-associated brain response phenotypes and mechanisms

### 3.1 Asthma and cognitive dysfunction

Clinical data indicate that approximately 45% of patients with asthma have varying degrees of cognitive impairment, which is closely related to the duration of asthma, frequency of attacks, and degree of pulmonary function reduction at the onset of asthma, and the risk of cognitive decline in patients with asthma increases by roughly 78% in comparison with the general population (8, 50, 51). Existing research has demonstrated that the precise mechanism by which asthma causes cognitive dysfunction is intricate, which is worthy of further study.

As mentioned previously, asthma can damage the BBB, allowing lung inflammatory factors to easily enter the hippocampus, PFC, and other regions of the brain associated with cognitive function, resulting in cognitive impairment. In addition, the onset of oxidative stress in asthma regulates the Th1/Th2-type immune inflammatory response, activates microglia, and promotes the release of inflammatory factors in cognitively involved brain regions such as the hippocampus by activating the NF-kB signal pathway, which may diminish N-acetylaspartate (NAA) levels in the hippocampus of patients with asthma and ultimately lead to cognitive dysfunction, including learning and memory (21, 52). Nair et al. noticed a high concentration of neurogranin (a protein closely related to synaptic plasticity and consolidation of memory and learning ability) and an elevated level of tau phosphorylation in the cerebrospinal fluid of patients with asthma, indicating that such patients have a pathological basis for cognitive impairment (53).

Given the contraction of bronchial smooth muscle and obstruction of airways, asthma attacks are accompanied by varying degrees of hypoxia symptoms, and the degradation of synaptic structures caused by hypoxia is closely associated with cognitive impairment (54, 55). Ren et al. revealed that the expression level of hypoxia-inducible factor-1α (HIF-1α) and HIF-2α was upregulated in the brain tissue of mice induced with asthma by house dust mites (HDM) along with the decreased synaptic plasticity in the hippocampus, and the learning and memory abilities of asthmatic mice, as measured by the Barnes maze test, were impaired compared with those of the control group (56, 57). In a mouse model of asthma, Guo et al. reported that the synaptic structure was destroyed in the hippocampus, and the maintenance of long-term potential (LTP), which is closely affiliated with learning, was disrupted, but this phenomenon did not affect the occurrence of LTP. In addition, the upregulation of HIF-1α expression and downregulation of c-FOS protein expression were detected in the hippocampus, and c-FOS is known to be a key factor in memory formation, that is, a strong correlation can be observed between hypoxia and cognitive impairment in patients with asthma (58). Moreover, cortisol hormones are used as conventional treatments to control asthma, and chronic oral cortisol hormones have been shown to promote volume reduction in the hippocampus and amygdala of patients with asthma (59). The aforementioned mechanism may be closely linked to cognitive decline in patients with asthma. However, recurrent asthma attacks; poor control often accompanied by sleep disorders, anxiety, depression, and other adverse emotional disorders; and sensitive responses to psychological stress and social stress events will damage patient’s learning and memory ability to varying degrees, which merits our focus.

Interestingly, in considering whether cognitive impairment has a reverse promoting effect on asthma pathogenesis, Wu et al. discovered evidence contradicting the hypothesis that inducing asthma in genetically inherited mice with Alzheimer’s disease (AD) did not exacerbate the severity of asthma, but rather reduced its airway hyperreactivity and airway obstruction, correspondingly increased the number of regulatory immune cells, and decreased the severity of inflammatory response to asthma (60). Contrary to the pathological progression of asthma, the existence of these protective mechanisms merits further investigation.

### 3.2 Asthma and anxiety disorders

Anxiety disorder is a mental illness characterized by excessive anxiety, worry, or dread. Asthma and anxiety disorders share many common predisposing factors, with an anxiety disorder incidence of approximately 22.7% and a risk nearly three times that of healthy individuals mentioned in a meta-analysis of comorbid anxiety in juvenile asthmatics (61). The co-occurrence of asthma and anxiety disorders diminishes the quality of life of patients; interferes with their ability to study, work, and engage in daily activities; makes asthma control more difficult; and increases the burden of medical care (62, 63).

Existing studies have indicated that the amygdala is a pivotal region of the brain encoding and processing anxiety-like behavior, and its circuit composed of mPFC plays a great part in the body’s anxiety processing network; moreover, the neural activity in this circuit can be directly used to represent the neuro-coding information of anxiety-like behavior (64, 65). Dehdar et al. characterized the immune-inflammatory status of the mPFC and amygdala in an ovalbumin (OVA)-induced asthma rat model by immunofluorescence staining of microglia and astrocytes and found that the activation and proliferation of these two cells were significantly enhanced probably because the immune-inflammatory factors produced during asthma inflammation strengthened the functional activity of local astrocytes via peripheral nerves or through the BBB to reach the paraventricular nucleus of the hypothalamus, stimulated their synthesis and release of gamma-aminobutyric acid, and induced the production of anxiety-like behavior, whereas the activation of microglia may heighten the inflammatory response in the hippocampus and further aggravate asthma-induced anxiety disorders (66–68). Further examination of the electrical activity of the brain in asthmatic rats revealed abnormal changes in the coupling potential between the mPFC and amygdala, such as decreased local delta/theta-gamma phase–amplitude coupling (PAC) in the mPFC, increased local delta-gamma PAC in the amygdala, diminished coupling between the mPFC delta/theta phase and amygdala gamma2 amplitude, enhanced coupling between the amygdala delta/theta phase and mPFC gamma2 amplitude, and improved neural activity in the mPFC and amygdala at low-frequency bands of delta and theta, indicating that chronic inflammation in asthma may achieve the neuromodulation of anxiety by strengthening the functional connectivity of the mPFC–amygdala circuit (69, 70). Dehdar et al. also found that inhaled corticosteroids, such as fluticasone, can improve the functional connectivity of the mPFC–amygdala pathway and attenuate the degree of neuroinflammation in the early stage of asthma pathogenesis, thereby relieving anxiety, which provides a foundation for the subsequent prevention and treatment of negative emotions in patients with asthma (70). More detailed studies have illustrated that ACC, as a subregion of the mPFC, also constitutes circuit connections with the basolateral amygdala (BLA) in a specific manner and plays an essential role in the neuromodulation of anxiety (71). Asthma-induced Th2 inflammatory factors allow type 2 cytokines to reach the ACC, BLA, and other brain regions along specific pathways, and the activation of microglia and astrocytes enhances the frequency of neural oscillations in ACC and BLA brain regions; moreover, the PAC of ACC-BLA circuits is strengthened, leading to heightened functional connectivity of ACC-BLA and inducing the expression of anxiety (72). In addition, anomalous coupling between the amygdala and the respiratory control network has a substantial effect on the development of anxiety in patients with asthma (73, 74). Hence, asthma regulates the development of anxious behavior in patients via numerous abnormal neural network activities.

Additionally, the expression level of type 2 inflammatory factors and corticotropin-releasing hormone was upregulated in the PFC, whereas c-FOS protein was deposited in the hypothalamus and amygdala of OVA-sensitized animal models, in which the activation of the HPA axis by corticotropin-releasing hormone could exacerbate asthma symptoms while modulating negative emotions (68, 75). Bejeshk et al. identified an oxidative/antioxidant imbalance in the hippocampus of asthmatic rats with anxiety, as well as elevated levels of inflammatory factors, and hypothesized that oxidative stress may have significant effects on asthma-related anxiety disorders, whereas the administration of antioxidant substances (e.g., myristicol) can ameliorate anxiety-like behavior by relieving inflammatory responses and oxidative stress in brain regions such as the hippocampus (76).

### 3.3 Asthma and depression

Epidemiological surveys have indicated that patients with asthmatic are roughly two times as likely as the general population to suffer from depression and that improvement in depressive symptoms contributes to the control of asthma progression (77). Th2-type immune inflammation in the pathogenesis of asthma can serve as a “link” between asthma and depressive disorders.

Accumulating research has verified that the NOD-like receptor family pyrin domain-containing 3 (NLRP3) inflammasome in lung-mediated pathological processes can promote a substantial increase in the serum concentration of pro-inflammatory cytokines (including IL-1β, IL-6, and TNF-α), corresponding activation of the HPA axis, and increased glucocorticoid release in patients with asthma. However, persistently high levels of glucocorticoids can lead to neuronal atrophy, neurogenesis inhibition, and decreased synaptic plasticity in the hippocampus and mPFC, whereas excessive IL-1β further reduces the production of brain-derived neurotrophic factor (BDNF) and neurogenesis in the hippocampus, both of which induce depressive mood in patients (78–80). Notably, Iwata et al. hypothesized that inflammatory signal transduction with the NLRP3 inflammasome as the “link” is biphasic in the development of depressive disorders in patients with asthma, that is, depressive symptoms among patients with asthma can be correspondingly activated by NLRP3 inflammasomes distributed in brain regions such as the hippocampus after pessimistic aggravation of major psychological stress events; the release of inflammatory factors such as IL-1β can transmit such inflammasomes to peripheral organs such as the lung through specific pathways (neural and humoral pathways) to strengthen the immune-inflammatory response of asthma, worsen the relevant symptoms of asthma, and serve as inflammatory factors in brain regions to further strengthen the susceptibility to depression along the abovementioned neural networks (81). However, direct experimental evidence is insufficient to confirm this biphasic regulatory relationship between asthma immune inflammation and neural networks in brain regions. If this hypothesis is validated, then it will have great guiding significance for the prevention and treatment of depressive disorders among patients with asthma.

In addition to inflammatory factors, Kanaya et al. found elevated levels of vascular endothelial growth factor (VEGF) in the blood of asthmatic mice and high concentrations of c-FOS protein, and glial fibrillary acidic protein (GFAP) deposition was detected in their brain regions, which is known to increase the permeability of the BBB. In addition, GFAP is a typical astrocyte injury marker, whereas c-FOS protein expression elevates when mast cells are activated in the brain, thereby indicating that inflammatory factors may infiltrate brain regions through the destruction of the BBB to activate neurons and mast cells in the brain, impair astrocyte function, disrupt mood regulation, and produce depressive disorders (82, 83). Depression and anxiety disorders, as common internalization disorders, involve many overlapping regulatory mechanisms in the pathogenesis of asthma, such as the activation of brain regions by oxidative stress, damage to brain structure by inflammatory factors, and feedback regulation of the HPA axis. Regulating these prevalent pathological processes will be more conducive to our prevention and treatment of asthma-related negative emotions (84).

### 3.4 Asthma and sleep disorders

Numerous studies suggest that patients with asthma frequently experience varying degrees of sleep disorders, which can manifest as insomnia, nocturnal awakenings, and early morning awakenings; indirectly contribute to daytime sleepiness, difficulty concentrating, diminished learning, and memory abilities; and significantly affect patients’ ability to work (85). What’s more, asthma is an essential risk factor for obstructive sleep apnea (OSA), with an incidence rate of up to 50% in patients with asthma, and the risk of OSA is proportional to the severity of asthma symptoms, the frequency of asthma attacks, and poor asthma control (86–88). Considering that asthma and sleep disorders can be affected by a variety of factors, such as obesity, smoking, rhinitis, and gastroesophageal reflux disease (GORD), the mechanisms involved are complex, and the role of “lung–brain crosstalk” remains unclear (89).

Bonnet et al. discovered significant increases in nocturnal airway hyperresponsiveness and airway resistance in patients with asthma, who tend to be agitated at night and experience more severe symptoms (90). Sensitizer exposure also caused a strong immune inflammatory response to worsen asthma symptoms, and Gervais et al. detected forced expiratory volumes in one second (FEV1) in patients with asthma after inhalation of HDM at different time points and found that FEV1 decreased evidently; moreover, ventilatory dysfunction was most severe after inhalation at 11 pm, which had a small effect at 8 am (91). The onset of asthma has a distinct circadian rhythm, which is closely linked to the development of sleep disorders in patients with asthma; however, the precise molecular mechanism remains unknown (92).

Asthma-related sleep disorders are inextricably linked to the expression of circadian clock-related molecules that modulate the circadian rhythm and activation of pathways (93). Ehlers et al. compared the broncholavage fluid from patients with severe asthma with controls and found that circadian clock-related genes (including BMAL1, PER2, and REV-ERBa) were all decreased and rhythm regulation may be disturbed (94). Zona et al. also discovered a dramatic increase in the number of eosinophils in the blood, the upregulation of the expression level of pro-inflammatory cytokines in the lungs, and a sharp increase in the degree of pulmonary inflammation in OVA-induced asthma in a BMAL1-knockout mouse model, which suggests that the regulation of circadian clock genes on asthma and rhythm may be biphasic (95). More in depth, rhythmic regulatory centers located in the suprachiasmatic nucleus (SCN) of the hypothalamus are stimulated after sensing afferent nerve impulses that activate the HPA axis and autonomic nervous system, and these nerve impulses include peripheral inflammatory molecules produced by asthma, which are transmitted to the brain through pathways such as the BBB, thereby resulting in the activation of brain regions or changes in brain function caused by the provocation of asthma by external stress factors (96, 97). This activity leads to the growing release of glucocorticoids and catecholamines (including epinephrine and norepinephrine), which can be further transmitted to the periphery to participate in the regulation of the immune inflammatory response to asthma, and the specific mechanism has been elaborated previously (98). Consequently, the modulation of circadian rhythm-related neural pathways in patients with asthma can not only reduce nocturnal sleep disorders, but also alleviate symptoms and produce the effect of one arrow and two sculptures.

### 3.5 Asthma and olfactory disturbances

Common chronic inflammatory diseases of the respiratory system, such as chronic rhinosinusitis (CRS) and AR, have been confirmed by many studies to be accompanied by varying degrees of olfactory dysfunction, which seriously impair the quality of life and mental health of patients, and such diseases are often complicated with asthma. Olfactory dysfunction caused by asthma is mostly induced on the basis of comorbidity with these diseases, and some scholars believe that asthma can be used as an independent risk factor for olfactory disturbance in patients with CRS (99, 100). Rhyou et al. conducted a clinical investigation on olfactory dysfunction in patients with asthma, which indicated that the incidence of olfactory disturbance in patients with CRS or AR associated with asthma was 56.5%, whereas the incidence of olfactory disturbance in patients with asthma alone was only 15.8% (10). Lacking direct research, the pathogenesis of asthma linked to olfactory dysfunction warrants further investigation.

**Figure 1:**
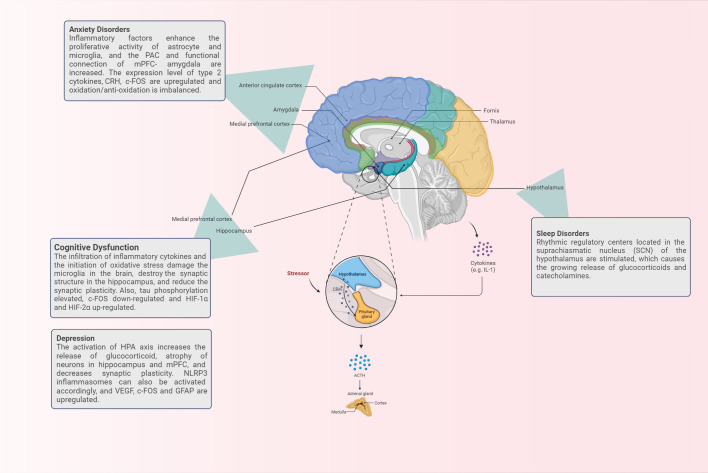
The brain regions and corresponding physiological mechanisms involved in the occurrence of brain response in asthma.

In summary, we have a preliminary understanding of the relevant brain regions and the pathological and physiological changes that occur when asthma patients are accompanied by cognitive dysfunction, anxiety, depression and sleep disorders, as shown in **Figure 1**. However, there is no unified theory that can fairly and comprehensively reveal its mechanism, which deserves further in-depth consideration.

## 4 Treatment of symptoms associated with brain response in asthma

**Figure 2:**
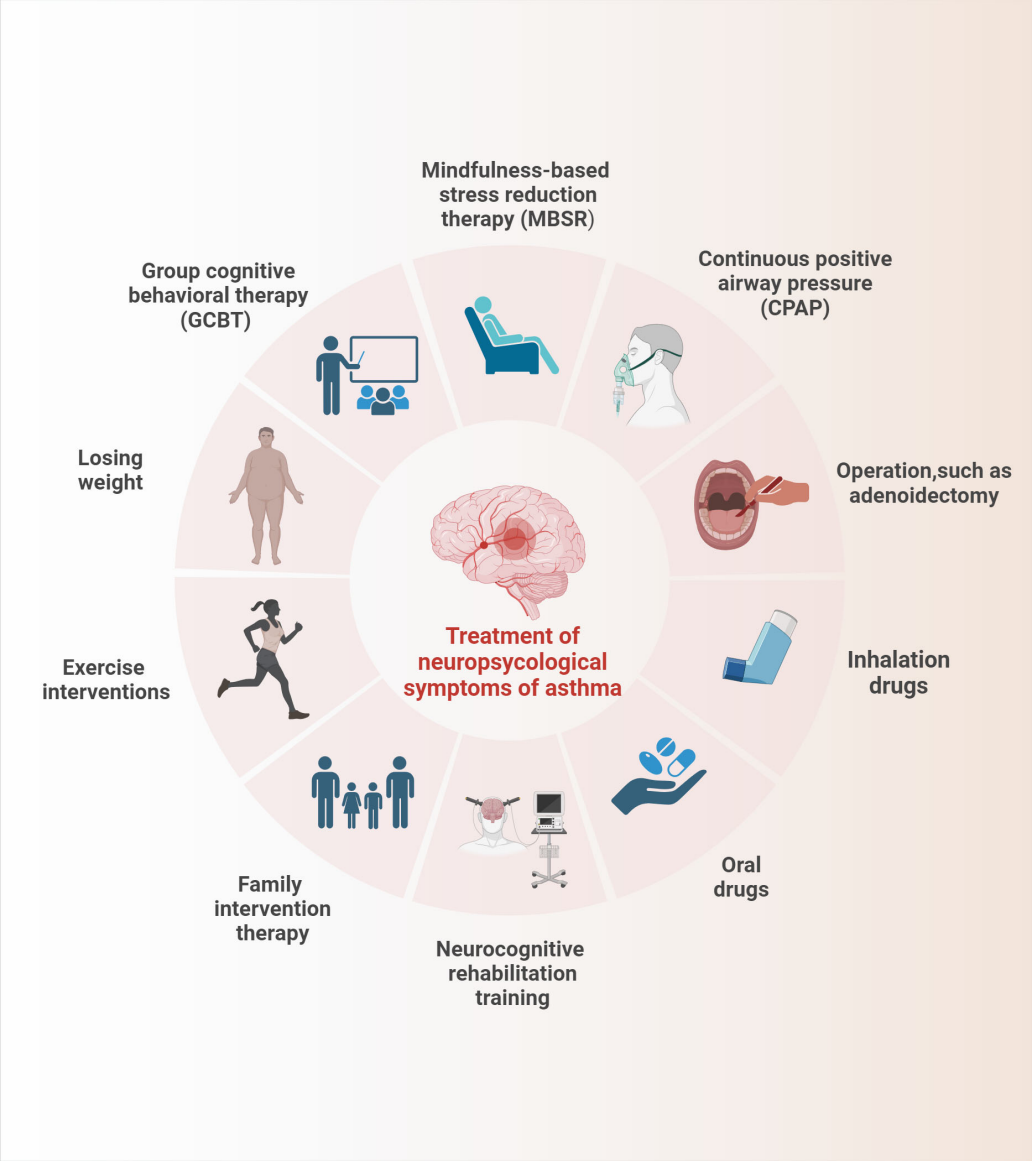
The summary of treatment of the neuropsycological symptoms in asthma.

Asthma causes cognitive impairment, depression, anxiety, and other emotional disorders as well as sleep disorders, olfactory dysfunction, and other neurological phenotypes, which will aggravate the psychological burden and life stress of patients with asthma while deteriorating the clinical symptoms of asthma, thereby resulting in a low quality of life. Consequently, while developing conventional medications for the treatment of asthma, a treatment to alleviate the neurological symptoms associated with brain response in patients with asthma must also be developed. At present, neurocognitive rehabilitation training, psychological intervention, continuous positive airway pressure (CPAP), exercise intervention, family intervention, and drug therapy are widely used and we have provided a brief summary in **Figure 2**. These interventions can be executed individually or in combination to alleviate each other’s symptoms, but their improvement of neurological asthma symptoms remains to be investigated (101).

Cognitive behavioral therapy (CBT), as one of the most common psychological interventions, is a psychotherapy designed to change a person’s thinking and behavior patterns through talking, which has been demonstrated to have good efficacy in a variety of psychological disorders (including cognitive impairment, anxiety, and depression) (102, 103). A clinical study based on group cognitive behavioral therapy (GCBT) revealed that GCBT can effectively rectify the functional connectivity activity between insular subregions and other brain regions in patients with asthma, which is essential for improving depressive symptoms (104).Of course, other psychological interventions, such as mindfulness-based stress reduction therapy, also have their exclusive effects in alleviating the neurological symptoms of asthma, but the direct effects of these treatments on the central nervous system remain to be studied (101).

Exercise interventions, such as common running, rope skipping, and other exercise modalities, can alleviate airway inflammation by balancing the ratio of Th1/Th2-type cytokines, reduce the response level of oxidative stress in the hippocampus to lighten oxidative damage, upregulate IL-10 levels in the hippocampus to strengthen brain inflammation, promote the induction and maintenance of LTP in the brain, and facilitate the synthesis and release of BDNF to increase synaptic plasticity, thereby relieving asthma-induced cognitive impairment (105–107). Concurrently, exercise can also play a certain role in weight loss. Obesity is known to be a risk factor for comorbid cognitive impairment and sleep disorders in asthma; thus, exercise is essential for neuroprotection (108).

Given its inhibition of GORD, local and systemic anti-inflammatory effects, enhancement of cardiac function, suppression of leptin levels, weight loss, and restoration of sleep patterns, CPAP, as a first-line treatment for OSA, has a substantial impact on the improvement of sleep disturbance in patients with asthma (109). Ninety-nine asthmatic patients with OSA treated with CPAP showed significant improvement in asthma symptoms and sleep disorders after 6 months, but evaluating the efficacy of CPAP treatment was exceedingly difficult because of the absence of a healthy control group (110). In addition, adenoidectomy combined with hormone inhalation, such as montelukast, can alleviate sleep disorders in asthmatic children with OSA (111).

Neurocognitive rehabilitation training refers to the intervention of related brain response to asthma by rehabilitation methods for conventional cardiovascular and cerebrovascular diseases, which only stays at the theoretical level and has not been reliably verified. Family intervention therapy begins with the mental health status of patients’ family members and seeks to alleviate related psychological disorders in patients with asthma by fostering a healthy family environment. Brown et al. suggested that a reduction in melancholy among family caregivers could effectively control the onset and progression of depression in children with asthma (112). By contrast, conventional drug therapy, which includes cortisol hormones, β-adrenergic agonists, etc., may have varying effects on the nervous system because of their specific modes, doses, frequencies, etc., and their benefits and drawbacks contradict the conclusions of the current study. For example, early inhalation of glucocorticoids, as mentioned previously, may alleviate the anxiety symptoms of patients with asthma, but their long-term use will worsen their cognitive impairment. Accordingly, additional multicenter, randomized, double-blind, parallel controlled studies must be conducted to investigate the duality of drug effects in depth.

## 5 Summary and prospect

**Figure 3:**
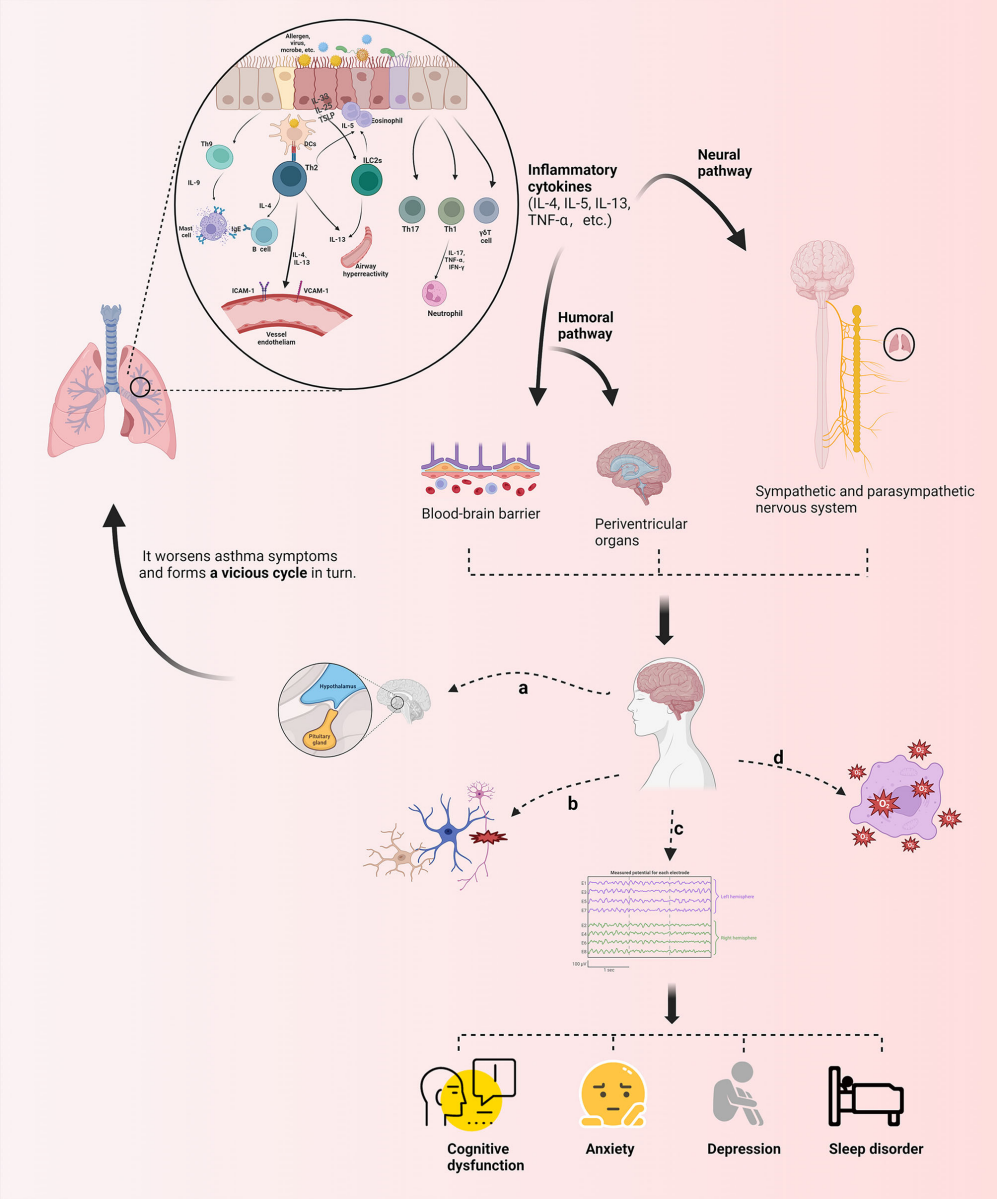
Schematic presentation of the mechanism of neurological phenotypes associated with asthma. Various immune inflammatory factors produced during the onset of asthma can reach the brain through humoral and neural pathways, inducing the production of corresponding brain response through different mechanisms, as follows: **(A)** Feedback regulation of the neuroendocrine axis (e. g) HPA) is activated; **(B)** Microglia, astrocyte, etc. are activated and proliferated, and neurons are damaged; **(C)** Brain wave amplitude, frequency, PAC between brain regions and potential are affected; **(D)** Imbalance in expression of oxidative and antioxidant molecules leads to changes in oxidative stress levels in brain regions. Theseneuropsycological symptoms further regulate the symptoms of asthma in turn, which forms a cycle. TSLP, the Thymic Stromal Lymphopoietin; ILC2s, group 2 innate lymphoid cells; IFN-γ, interferon-gamma; ICAM-1, intercellular adhesion molecule 1; VCAM-1, vascular cellular adhesion molecule-1 (The figure is created by BioRender.com).

Through a systematic exposition of the incidence condition, the inflammatory factors and neural circuits involved, and corresponding treatment methods of neuropsycological symptoms in asthma, we recognize that its pathogenesis is complex and may be the result of a combination of multiple physiological and pathological mechanisms. Furthermore, we summarize its mechanism diagram in **Figure 3**: (1) A variety of cytokines are produced during the inflammatory response to asthma afferent the nervous system through the humoral pathway or neural pathway, of which the humoral pathway refers to the penetration of inflammatory factors through the BBB or periventricular organs without the BBB into the brain region. In addition, the neural pathway indicates the activation of sympathetic, parasympathetic, and sensory nerves via immune inflammatory factors; the release of multiple neuropeptides and neurotransmitters; and the excitation of the corresponding electrical signals to change the stimulation of the specific brain regions. (2) Inflammatory infiltration in the brain region causes the activation and proliferation of microglia and astrocytes as well as damages neurons, resulting in changes in the corresponding electrical activity of the brain, of which the brain wave amplitude, frequency, PAC between brain regions, and potential are affected. The feedback regulation of the neuroendocrine axis is also initiated accordingly, which in turn acts on related effectors in the lungs and worsens asthma symptoms. (3) Neuroinflammation transmitted to the brain influences the level of oxidative stress in the brain, and the equilibrium between oxidative and antioxidant molecules is disrupted, resulting in functional changes in specific brain regions. (4) Whether the relationship between asthma and olfactory dysfunction is mediated or directly related by nasal diseases lacks relevant evidence. A summary of changes in brain network/activity, mediators, and related brain regions involved in the response of the brain in asthma is shown in **Table 2** (12, 21, 31, 34, 37, 42, 44, 49, 53, 56, 58, 59, 68, 70, 72, 74, 78, 82, 97, 113, 114).

**Table 2:** Summary of mediators, brain regions, brain networks, and brain activities involved in the development of brain responses to asthma.

Brain Response	Mediators	Associated brain region	Associated brain network/brain activity	References
Multiple neurological phenotypes (Not specified)	CCL11(eotaxin-1)	Microglia and astrocyte in the brain.	CCL11 triggers oxidative stress via microglial NOX1 activation and potentiates glutamate-mediated neurotoxicity.	Parajuli B, et al. (31)
Multiple neurological phenotypes (Not specified)	Leukocytes	The cerebral cortex.	The recruitment of leukocyte increases the level of oxidative stress in cerebral cortex and induces neuronal damage.	Antunes GL, et al. (113)
Multiple neurological phenotypes (Not specified)	Neural impulses transmitted by pulmonary receptors.	The NTS.	The plasticity of postsynaptic neurons increases due to variations in the amplitude and frequency of nerve impulses transmitted to the NTS by lung receptors.	Chen CY, et al. (37)
Multiple neurological phenotypes (Not specified)	Airway chemoreceptors and pulmonary stretch receptors Information.	ACC, insula, the brainstem, the prefrontal cortex and spinothalamic cortex.	Functional connectivity is formed between brain regions to regulate the respiratory network and change the respiratory pattern.	Vafaee F, et al. (12)
Multiple neurological phenotypes (Not specified)	IgE and NE.	Hypothalamus and pituitary body.	The hypothalamus activates the sympathetic nervous system through the HPA axis to release NE, which promote B cell to release IgE.	Pongratz G, et al. (44)
Multiple neurological phenotypes (Not specified)	IL-5, GM-CSF, TNF-α, IL-6 and glucocorticoids.	Hypothalamus and pituitary body.	After activation of the HPA axis, glucocorticoids are released, leading to downregulation of GR in the lung and a decrease in the body’s responsiveness to glucocorticoids.	Bailey MT, et al. (42)
Multiple neurological phenotypes (Not specified)	Histamine	The cerebral cortex.	Allergens under stress may increase the severity of the inflammatory response to asthma by stimulating the release of histamine to activate histamine receptors (H1R and H2R) in the brain region.	Miyasaka T, et al. (49)
Cognitive dysfunction	Inflammatory cytokines	The hippocampus.	Inflammatory factors activate microglia, promote the release of inflammatory cytokines in hippocampus, and lead to lower levels of NAA and Glu in hippocampus.	Kroll JL, et al. (21)
Cognitive dysfunction	Not mentioned.	Synaptic structure in cerebral cortex.	Synaptic degeneration biomarkers neurogranin and α-synuclein increase and the degree of tau phosphorylation elevates.	Nair AK, et al. (53)
Cognitive dysfunction	IL-4, IL-5, and TNF-α.	The cerebral cortex and hippocampus.	Inflammatory infiltration causes cerebral vascular edema, up-regulation of HIF-1α and HIF-2α expression in brain tissue, and decreased synaptic plasticity in the hippocampus.	Ren M, et al. (56)
Cognitive dysfunction	Inflammatory cytokines	The hippocampus.	Synaptic structure and maintenance of LTP were disrupted in the hippocampus, while the expression of HIF-1α was up-regulated and the expression of c-FOS protein was down-regulated.	Guo RB, et al. (58)
Cognitive dysfunction	Cortisol hormones	The hippocampus and amygdala.	Chronic use of cortisol hormones results in greatly reduced volumes of the hippocampus and amygdala.	Brown ES, et al. (59)
Cognitive dysfunction and anxiety disorders	IL-6, IL-17 and TNF-α	The hippocampus	Inflammatory infiltration in the hippocampus decreases levels of oxidative molecules, increases levels of antioxidant molecules, oxidative/antioxidant imbalance, and enhanced oxidative stress.	Bejeshk MA, et al. (76)
Anxiety disorders	IL-9, IL-13, IL-1β and eotaxin.	The hypothalamus.	Inflammatory factors alter the activities of AchE and Na +, K + -ATPase in brain regions, resulting in increased ROS levels, up-regulation of BDNF expression and down-regulation of GR expression.	Antunes GL, et al. (34)
Anxiety disorders	Inflammatory cytokines	The hypothalamic paraventricular.	Inflammatory factors reach the hypothalamic paraventricular nucleus to activate astrocytes and microglia, promote their release of γ-aminobutyric acid, and enhance the inflammatory response in the hippocampus.	Dehdar K, et al. (68)
Anxiety disorders	Inflammatory cytokines	The mPFC and amygdala.	Inflammatory activation in brain regions resulted in abnormal changes in coupling potentials between the mPFC-amygdala, with mPFC-amygdala delta/theta-gamma PAC weakened, and amygdala-mPFC delta/theta-gamma PAC enhanced.	Dehdar K, et al. (70)
Anxiety disorders	IL-13, TNF-α, etc.	The ACC and BLA.	The arrival of inflammatory factors in brain regions increases the frequency of neural oscillations in ACC and BLA, enhances PAC and functional connectivity in ACC-BLA circuits and disrupts top-down and bottom-up regulation.	Gholami-Mahtaj L, et al. (72)
Anxiety disorders	Inflammatory cytokines	The ARA loop.	Inflammation in brain regions decreases synchrony of the amygdala with respiratory rhythms, disrupts the ARA circuit, and increases local phase power coupling between δ-γ2 and θ-γ2 in the amygdala.	Dehdar K, et al. (74)
Anxiety disorders	IL-4, IL-5 and IL-13.	The hippocampus and brainstem.	After the airway inflammation, brainstem SERT mRNA, hippocampal 5Htr1a and Crhr1 expression are all ug-regulate.	Caulfield JI, et al. (114)
Depression disorders	IL-1β, IL-6, TNF-α and NLRP3 inflammasome.	The hippocampus, mPFC and HPA axis.	NLRP3-mediated inflammation in the lung activates the HPA axis, and excess glucocorticoids released can lead to neuronal atrophy and reduced synaptic plasticity in the hippocampus and mPFC, while excess IL-1β further reduces BNDF production and neurogenesis in the hippocampus.	Ma M, et al. (78)
Depression disorders	Pro-inflammatory cytokines and Th2-related cytokines (IL-4, IL-5, IL-13, etc.)	The cerebral cortex.	Inflammation in the lung may lead to up-regulation of VEGF, c-FOS protein and GFAP expression in brain regions, and mast cell activation in brain regions may mediate the generation of depression-like behavior by affecting the function of related brain regions.	Kanaya A, et al. (82)
Sleep disorders	Pro-inflammatory cytokines	The SCN of the hypothalamus.	Rhythm regulatory centers located in the SCN are activated upon sensing afferent nerve impulses from pulmonary inflammation, activating the HPA axis and autonomic nervous system, releasing glucocorticoids and other worsening asthma symptoms.	Mavroudis PD, et al. (97)

NE, norepinephrine; Glu, glutamate; GM-CSF, granulocyte-macrophage colony-stimulating factor; ARA, amygdala–respiration–amygdala; SERT, serotonin transporter; 5Htr1a, serotonin receptor 1a; Crhr1, corticotropin releasing hormone receptor 1.

It cannot be ignored that not all cognitive dysfunction and mood disorders (anxiety, depression) associated with asthma patients are positively correlated with the level of pulmonary inflammation. For example, asthma in elderly patients was negatively correlated with the degree of cognitive impairment in a study of asthma patients of different ages by Minna et al. and others have found a strong gender bias in the distribution of asthma patients with anxiety, with significantly more males than females and a positive correlation with age (115, 116). More detailed studies have shown that asthma control and the degree of airway obstruction during exacerbations do not significantly associate with cognitive impairment in elderly patients, and we hypothesize that the inflammatory state of the body in asthma patients may be a contributing, rather than a primary, factor in the development of cognitive deficits in the elderly, but there is a lack of direct evidence to prove this (117). In addition, in a study comparing anxiety and depression symptoms in children with asthma before and after hospitalization, it was noted that more than half of the children had a significant increase in anxiety and depression, or even a combination of both, and that nearly 26% of the children showed a persistent increase in adverse moods at discharge follow-up after well controlled of lung symptoms. It does not seem to be fully explained by the theory of the “lung-brain” axis mediated by the neuroimmune crosstalk mechanisms that we have summarized 4and may be associated with the patient’s own degree of sensitivity and tolerance to the perception of symptoms such as wheezing, also known as symptom-related anxiety/depression (118, 119). To some extent, this suggests that the neuropsychological symptoms induced in asthma patients may depend not only on the severity of the asthma attack, which may reflect the level of inflammation in the body, but may also be related to the delayed response of the body to the deterioration of the disease, the stress of hospitalization, and other stressful factors, that is to say, post-traumatic stress disorder (PTSD) cannot be ruled out, especially in the context of the Corona Virus Disease 2019 (COVID-19) pandemic, the influence of the superimposed effect should not be ignored (120). Thus, it is clear that multiple factors other than neuroimmune mechanisms are involved in the development of asthma-associated neuropsychological symptoms, which deserves further investigation.

The “lung–brain” crosstalk phenomenon between asthma and its brain response is governed by a biphasic regulatory mechanism, that is, the inflammatory response caused by asthma is transmitted to the central and peripheral nervous systems through the abovementioned pathways, which stimulates the synthesis and release of neurotransmitters such as neuropeptides, which in turn can further bind to the corresponding receptors on the airway surface to promote inflammatory progression and worsen asthma symptoms. Unique occurrences, such as asthmatic mice, accompanied by cognitive impairment, alleviate the inflammatory response to relieve the symptoms of asthma. This review will encourage more researchers to focus on the brain response and its mechanism in asthma, and more in-depth studies can be conducted on this topic to provide additional evidence for guiding the diagnosis and treatment of asthma using the “lung–brain” axis theory and new hope for enhancing the quality of life of patients with asthma.

## Author contributions

YW, Y-KM and H-RW contributed equally to this work. X-CS and CR: conception, design and administrative support. YW, Y-KM and H-RW: provision of study materials. YW, Y-KM and H-RW, X-YS and S-ZW: collection and assembly of data. YW, Y-KM and H-RW, CR and X-CS: data analysis and interpretation. All authors: manuscript writing and final approval of manuscript.

## Glossary

**Table:**

AR	allergic rhinitis
fMRI	functional magnetic resonance imaging
PAG	periaqueductal gray
dVMHC	dynamic voxel-mirrored homotopic connection
NO	nitric oxide
BBB	blood–brain barrier
CCL11	eotaxin-1
NOX1	nicotinamide adenine dinucleotide phosphate-oxidase 1
ROS	reactive oxygen species
AchE	acetylcholinesterase
Ach	acetylcholine
NTS	nucleus tractus solitarius
ACC	anterior cingulate cortex
mPFC	medial prefrontal cortex
LTA	life-threatening asthma
PFC	prefrontal cortex
HPA	hypothalamic–pituitary–adrenal
GR	glucocorticoid receptors
IgE	immunoglobulin E
SP	substance P
NPY	neuropeptide Y
NAA	N-acetylaspartate
HIF-1α	hypoxia-inducible factor-1α
HDM	house dust mites
AD	Alzheimer’s disease
OVA	ovalbumin
PAC	phase–amplitude coupling
BLA	basolateral amygdala
NLRP3	NOD-like receptor family pyrin domain-containing 3
BDNF	brain-derived neurotrophic factor
VEGF	vascular endothelial growth factor
GFAP	glial fibrillary acidic protein
OSA	obstructive sleep apnea
GORD	gastroesophageal reflux disease
FEV1	forced expiratory volumes in one second
SCN	suprachiasmatic nucleus
CRS	chronic rhinosinusitis
CPAP	continuous positive airway pressure
CBT	cognitive behavioral therapy
GCBT	group cognitive behavioral therapy
